# Critical success factor based resource allocation in ERP implementation: A nonlinear programming model

**DOI:** 10.1016/j.heliyon.2022.e10044

**Published:** 2022-07-25

**Authors:** Ying Xie, Colin Allen, Mahmood Ali

**Affiliations:** a Faculty of Business and Law, Anglia Ruskin University, Bishop Hall Lane, Chelmsford, CM1 1SQ, UK; b Faculty of Business, University of Greenwich, UK; c University of Business and Technology, Jeddah 21361, Saudi Arabia

**Keywords:** ERP implementation, SMEs, Critical success factor, Resource allocation, Nonlinear programming

## Abstract

This research examines how a Constrained Nonlinear programming model for ERP implementation (CNL_ERP) can facilitate Small and medium sized enterprises (SMEs) to deploy resources to address the Critical Success Factors (CSFs) in the pre-implementation phase, and to invest in them during implementation to increase the probability that the implementation will be successful. Applications of CNL_ERP in three case studies demonstrate that the average ERP implementation outcomes outperform the observed results. Using the Generalised Reduced Gradient Method, we developed an ERP implementation strategy realising resource allocation to CSFs. The strategy provides a rich picture of where to concentrate effort in the initial, intermediate and final phases, and is very helpful in enabling an SME to understand the progress of an ERP project and the resources needed. In case there are changes in resources (such as budget, team performance), the model enables SMEs to rank CSFs, and to adjust resources allocations accordingly to achieve the best ERP implementation performance.

## 1 Introduction

An Enterprise Resource Planning (ERP) implementation project includes three phases (Hasibuan and Dantes, 2012): (1) pre-implementation, (2) implementation, (3) post implementation. Preparing for the project in the pre-implementation phase is crucial to ensure successful implementation of ERP (Sun et al., 2016; Jagoda and Samaranayake, 2017). In order to implement a successful ERP project an organisation will have to acquire adequate levels of employees' skills, vendor support and resources in the pre-implementation stage (Ahmadi et al., 2015), and to deploy resources optimally to address critical success factors (CSFs) in the implementation stage (Ahmadzadeh et al., 2021).

ERP implementations in SMEs are especially vulnerable due to three challenges: 1) an ERP project is complex and large scale; 2) the schedule of the project is usually tight due to competitive pressures; 3) SMEs have limited resources to devote to the implementation, and these resources have limited or zero prior knowledge or experience relating to an ERP system. The emergence of Cloud based ERP systems has enabled SMEs to experience the advantages of an ERP package while decreasing the upfront costs of computing infrastructure and required IT support (Fosso-Wamba et al., 2015). To implement a Cloud based ERP, SMEs depend heavily on the services and support offered by a Cloud vendor to organise data and update software (Hashem et al., 2015). However, a Cloud based ERP solution does not remove the key requirements for a successful ERP implementation. The CSFs that can help an SME achieve a successful Cloud based ERP implementation (Ahmad and Mehmood, 2016) are similar to those identified for on-premises ERP implementation. Past research has attempted to examine the associations of CSFs with ERP project success using quantitative or qualitative methods, such as multicriteria decision making, interviews or surveys. However, empirically testing the effectiveness of CSFs on ERP project success will contribute significantly to the existing body of knowledge (Kirmizi and Kocaoglu, 2022).

In addition to understanding the effectiveness of CSFs on ERP project success, the ERP project manager needs to know which CSFs should be prioritised at each stage and how to allocate limited resources to address them (Sun et al., 2015; Kirmizi and Kocaoglu, 2022). The lack of quantitative measurement of CSFs' performance and their contributions to overall ERP implementation performance has led to a fragmented and partial understanding of how to address the selected CSFs in order to achieve a successful implementation of, and performance improvement from, ERP projects.

SMEs are recommended to acquire resources to maximise the degree of readiness prior to implementation (Ahmadi et al., 2015), and to invest in them during implementation so as to improve the chances of successful implementation (Saade and Nijher, 2016). Resource allocation has been widely discussed in other sectors, such as in communications (Zhou et al., 2016) and edge computing (Liao et al., 2020), where quantitative algorithms and frameworks were developed to allocate power supplies and computational tasks. However, literature pertaining to resource allocation in ERP implementation is scarce, with a focus technical and social processes (Phillips and Bana e Costa, 2007) or key resources required (Chatti et al., 2021). While some sources identify resource allocation as an important ERP adoption factor (Verdouw et al., 2015), very few ERP studies determine or estimate quantitatively the resources required to achieve target ERP performance.

This research attempts to fill the research gaps, by a) associating CSFs with ERP implementation performance using quantitative methods, b) quantifying resource allocation to address CSFs in ERP implementation, and c) empirically testing and validating the effectiveness of investment in CSFs on ERP project success. As only a limited number of SMEs have adequate resources to adequately address all the CSFs (Sun et al., 2015), this research aims to answer two research questions: (I) How can SMEs achieve target ERP implementation performance by appropriately allocating resources, i.e., time and budget, to address CSFs? (II) In response to changes in resources available for ERP implementation (such as budget, team performance), how can SMEs adjust resources allocated to address CSFs so as to optimise ERP implementation performance? This study is based on a combination of analytical modelling and empirical case studies, demonstrating the practical application of CNL_ERP.

The paper is organised as follows. The relevant literature is reviewed in Section 2. In Section 3, through a combination of modelling and empirical surveys, CNL_ERP is developed, combining both analytical regression models and constrained nonlinear programming models. Section 4 shows the application of CNL_ERP via case studies. Finally, theoretical and managerial contributions, and directions for further research are presented in Section 5.

## 2 Literature review

The implementation stage of ERP has been widely studied, including CSF identification, strategies and approaches for implementation, knowledge transfer and organisational ERP fit. While it is often argued that the implementation of ERP is a continuous cycle of improvement, the parameters of this investigation are limited to ERP implementation post ERP software selection and project planning. This research develops a tool to forecast the resources, i.e. project schedule and budget allocation (Nagpal et al., 2015), required for implementing ERP from the initial training until the desired ERP implementation performance level is achieved. In this section, we review the CSFs of ERP implementation and various ERP performance measures, as well as the quantitative models developed for ERP implementation.

### 2.1 Critical success factors

The discussion of CSFs is a predominant research stream in ERP literature, even in the era of Cloud computing, which has resulted in a shift of ERP systems to Cloud platforms (Fosso-Wamba et al., 2015). Cloud-based solutions remove the requirements to install IT hardware on premises and to maintain an IT workforce in organisations, making ERP implementation more affordable for SMEs. Recently, more research has been carried out to understand the impact of CSFs on ERP implementation performance, for both Cloud-based and on-premises ERP solutions (Alharthi et al., 2017; Ahn and Ahn, 2020; Gupta and Misra, 2016). Traditional CSFs relating to both organisational and technical aspects prove to significantly impact the successful implementation of Cloud-based ERP (Gupta et al., 2018), including top management support, project management, change management, business process reengineering (Vargas and Comuzzi, 2020; Malik and Khan, 2020), user training and education, clear objective setting, and interdepartmental communication (Tarhini et al., 2015). The importance of, and associations between, these CSF factors were further verified by Baykasoğlu and Gölcük (2017) using interpretive structural modelling and fuzzy cognitive maps.

The other significant development relates to attempting to associate CSFs with the implementation stages. Drawing on real world case studies, Saade and Nijher (2016) consolidated a list of CSFs during the ERP implementation process and related these to the five ERP implementation stages. To manage the performance of CSFs in each ERP stage, Sun et al. (2015) proposed performance assessment models and developed KPIs for CSFs. The model developed by Sun et al. (2015) has the functionality to quantitatively measure ERP project performance at each stage and to identify remedial actions if the performance falls below expectation; therefore, it could serve as a tool to decide where and when during the ERP lifecycle a CSF should be applied.

While there has been plenty of research exploring the CSFs for ERP implementation, and associating CSFs with ERP implantation stages, research into how to address, resource or administer CSFs during ERP implementation has been limited. By combining an empirical survey and mathematical modelling, we aim to provide both empirical and scientific evidence of the direct influences of the chosen CSFs on ERP implementation performance. Resource allocation is a key element of the ERP implementation strategy (Parr and Shanks, 2000), and is the focus of this research. Since this research focuses on ERP implementation after ERP software selection and project planning, we do not consider factors in relation to ERP software selection, organisational environment, organisational experience or change management. We have chosen to examine how to address five CSFs in an ERP implementation project, including Top Management Support (TM), Users, IT Infrastructure (IT), Project Management (PM), and Vendor Support (VS) (see Table 1). Despite the terminological differences around CSF names that exist in the literature, the attributes considered under these five CSFs represent a comprehensive list of factors that are identified as being directly associated with ERP implementation stages and having an important influence on the success of ERP project delivery (Sun et al., 2005). We chose these five CSFs for the following reasons.

• The five chosen CSFs are oriented towards the implementation stage (Vargas and Comuzzi, 2020; Gupta et al., 2018); therefore, they fit well with the aim of this study.
• These five CSFs have been consistently categorised and highlighted as important factors for successful implementation of ERP projects (see references in Table 1).
• These CSFs have not previously been examined with regard to their association with key measures relating to project deliverables and constraints, including cost, time and contribution to the overall ERP implementation performance.

**Table 1:** Five Critical Success Factors considered in this research.

CSFs identified for ERP implementation	CSF Attributes	References that have identified the CSF
*CSF*_1_ - Top Management support (TM)	Leadership, participation and commitment of the senior level of management	Tarhini et al., 2015; Malik and Khan, 2020
*CSF*_2_ - Users	Users' perception, interest, commitment, participation, feedback, IT skills, team dynamics, in house training, familiarity with other team members and external consultants	Plaza et al., 2010; Baykasoğlu and Gölcük, 2017
*CSF*_3_ - IT infrastructure (IT)	Hardware, software and IT architecture, databases of appropriate quality and data migration capability	Gupta et al., 2018
*CSF*_4_ - Project Management (PM)	Project team selection, team training, team competence, project tracking, business process reengineering	Baykasoğlu and Gölcük, 2017; Gupta et al., 2018; Vargas and Comuzzi, 2020
*CSF*_5_ - Vendor Support (VS)	Vendor expertise relating to training, technical knowledge and support, maintenance, emergency management, updates, service responsiveness and reliability	Malik and Khan, 2020

### 2.2 ERP implementation performance

ERP implementation performance can be defined by multiple aspects, depending on when the performance is measured and who measures it. The project manager's key objective is to deliver the project on time and within budget while, at the organisational and user levels, the aim is to reap the projected operational benefits of the ERP system (Kirmizi and Kocaoglu, 2022). The performance of implementing ERP as an information system is usually measured at the end of the go live stage, and based upon project delivery outcomes (Ram et al., 2013). Such performance is defined by multiple parameters, such as time, cost and functionality, to assess whether the expected objectives are being achieved through the implementation within the limitations (Lima et al., 2013; Sun et al., 2016). The main functionality expected from an ERP implementation is to enhance integration of commercial processes in an organisation and, consequently, to deliver the projected operational benefits, including improved efficiency, reduced production costs and maximised profits (Kirmizi and Kocaoglu, 2022). The percentage of such functionality achieved by the ERP implementation is also an important evaluation criterion.

ERP implementation performance has been researched as a qualitative measurement or as a quantitative measurement, and measured subjectively, by either experts or users using measurement scales. For example, Zareravasan and Mansouri (2016) quantitatively measured the outcome of ERP projects using budget, time, and user expectations. Sun et al. (2015) measured performance at each stage using CSF weighted KPI scores and identified remedial actions if the performance was below expectation. Plaza and Rohlf (2008) and Plaza et al. (2010) defined performance as the rate of competition of a task (the number of modules configured, or the number of transactions completed), and modelled the performance as a mathematical function that is dependent on time, training and learning. Sun et al. (2005) developed a quantitative evaluation of overall ERP implementation performance in terms of utilisation of the ERP system's capabilities, and the organisation's functionality requirement that is met by the ERP system.

In this study, ERP implementation performance is defined from the system implementation point of view and evaluated as to whether the project is completed within time and budget limitations, and whether the adopted ERP system helps the SME achieve the required level of effectiveness (Bhatt et al., 2021). A quantitative evaluation of the performance level is defined as the percentage of the organisation's target functional requirements met by the ERP implementation. The overall success of ERP implementation relies on the degree to which CSFs are addressed during implementation; therefore, the evaluation of overall ERP implementation performance level, cost and time is broken down by CSFs.

### 2.3 Quantitative models on ERP implementation

Many researchers investigated the direct association between CSFs and ERP implementation performance (Gupta et al., 2018; Malik and Khan, 2020). Traditionally, qualitative research has been the most prevalent research method in studying such relationships. While the identified CSFs in case studies or surveys enable SMEs to develop a better understanding of the CSFs' impacts, the impacts extent is unclear, limiting the ability of SMEs to make effective ERP implementations interventions based on the research. A scientific model is needed to suggest how, when and which CSFs should be addressed during ERP implementation so that organisations can plan and execute ERP projects that result in a more successful implementation (Vargas and Comuzzi, 2020).

The use of operational research (OR) approaches to research ERP implementation has also received more attention over the last decade. OR models have the ability to evaluate existing concepts of ERP as well as evaluating and setting critical success strategies for ERP projects (Yeh and Xu, 2013). For example, Plaza and Rohlf (2008) investigated how the training, learning and performance of the project team can minimise ERP project consultancy costs, and developed an analytical model to predict the project completion date. Based on their 2008 work, Plaza et al. (2010) presented a comparative analysis of two types of learning curves and illustrated how they can be applied in four ERP implementation projects. Although Plaza and Rohlf's work enhanced the traditionally qualitative ERP research by developing quantitative models, their work has certain limitations: 1) only one CSF, project team progress, is addressed; 2) the analytical models developed are only tested in the context of the case study organisations and not validated by statistical analysis; 3) analytical models cannot provide dynamic views on the ERP implementation project processes.

Complementing, and in contrast to, research that utilises deductive research approaches to develop quantitative modelling, Sun et al. (2005) used realistic data to quantify the measurements to be addressed for CSFs during ERP implementation, including cost, schedule, and goal achievement. They also developed a simulation model to help SMEs develop appropriate measurements to measure ERP implementation achievement. However, their model lacks key functionality relating to predicting the resources needed by SMEs and cannot help SMEs to plan resources in advance. As a result of restricted resource availability, Sun et al. (2005)'s simulation model was limited with regard to both validity and generality as: 1) only 6 SMEs were observed; 2) other data were generated using a data fitting method; and 3) it was assumed that variations between observed data and generated data were insignificant.

Xie et al. (2014) developed an integrated decision support system (DSS) for ERP implementation in SMEs, combining logistic regression models, linear regression models, a nonlinear programming model, and a simulation model, to predict ERP project implementation outcomes and facilitate the allocation of resources. However, the validity of the DSS needs to be further tested in empirical studies to ascertain its practical use and benefits. The robustness of the model also needs to be tested and analysed.

Zareravasan and Mansouri (2016) proposed a fuzzy cognitive map based dynamic model of ERP failure factors through project lifecycle phases. Imitating human reasoning, this tool models uncertainty and related events and could be used to assess the joint influence of ERP implementation failure factors on project outcomes. However, this tool is limited to the Iranian context and generalisation would be difficult. The model also strongly depends on experts' subjective judgement on the interrelationships between factors; hence, both its use and its outcomes could vary significantly with different groups of experts.

The above review indicates that mathematical programming and simulation have been valuable in providing insights into specific problems, facilitating organisational preparation for ERP implementation, and achieving success in ERP implementation; however, a mathematical model that proves to be beneficial to firms in identifying required resources and in developing an implementation strategy realising resource allocation, has not been available. The current research aims to fill this gap in the literature and takes the view that the decision to allocate resources (time and budget) to address CSFs should be made by considering resource constraints with the aim of maximising ERP implementation performance.

## 3 Mathematical models

**Figure 1:**
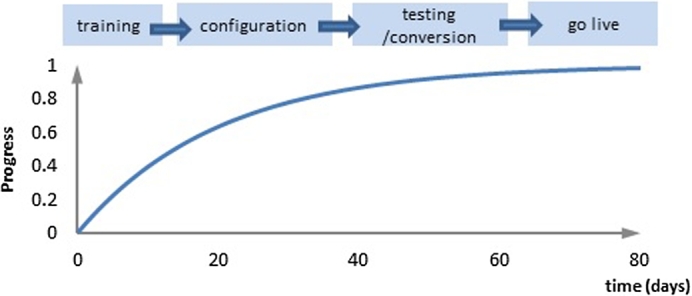
A logistic curve for an ERP implementation project.
• Performance level: denoted by *PF*, the percentage of the organisation's expected functional requirements met by the ERP implementation.

Mathematical models were constructed to show the relationships between implementation cost and project duration, as well as the ERP implementation performance level over the project duration. Three important parameters were introduced in the models:

• Implementation cost: denoted by *C*, the cumulative cost of the overall ERP implementation project.
• Project duration: denoted by *T*, the time elapsed from the start of the initial training phase to the final go live phase, covering the configuration, testing, and conversion phases (see Fig. 1).

The following notations are used to model parameters and variables:

*M* total number of CSFs considered,
*i* subscript of a CSF, i=1,2,…,M,
$d_{i}$ coefficient of the cost function,
$t_{i}$ time spent on the ${\mathrm{CSF}}_{i}$,
${\mathrm{cost}}_{i}$ cost consumed by ${\mathrm{CSF}}_{i}$,
${\mathrm{PF}}_{i}$ progress made by ${\mathrm{CSF}}_{i}$,
$p_{i}$ performance threshold of ${\mathrm{CSF}}_{i}$,
$k_{i}$ progressing curve coefficient of ${\mathrm{CSF}}_{i}$, measuring progressing speed of ${\mathrm{CSF}}_{i}$,
*T* project duration of an ERP implementation,
*PF* performance level achieved in an ERP implementation,
*C* cost consumed by an ERP implementation,
*TL* limitation on ERP project duration,
*CL* limitation on ERP implementation cost (budget),
$g_{1}$ ERP project duration, imposed as constraint 1 on CNL_ERP,
$g_{2}$ ERP implementation cost, imposed as constraint 2 on CNL_ERP
*PI* proximity index.

### 3.1 Assumptions

In order to model a near to reality ERP implementation in relatively simplified mathematical models, and to focus on the resource allocation for ERP implementation, we introduce several assumptions:

Assumption 1 Teams are fully prepared for ERP implementation and have received the necessary briefings and consultation to understand the vision behind the ERP implementation, the basis of the ERP system, the required changes, and the predefined implementation strategy.

Assumption 2 Organisations have been given intensive training and education to upgrade skills relevant to the ERP software and there is support from the vendor. Due to this training, teamwork and collaboration have been achieved and organisations are ready for ERP implementation; therefore, we can ignore the start-up effect in the ERP project implementation.

Assumption 3 The dynamic ERP implementation environment can be quantified and modelled using mathematical models at CSF level.

Assumption 4 The internal costs of ERP project implementation, including the one-off purchase of ERP software and hardware, overhead costs, and system installation costs are not considered in the mathematical model. The model only considers dynamic cost, which changes with the time spent to address each CSF.

### 3.2 Modelling performance against time using the logistic curve

The S-Curve is the one most commonly used project management tools for cost estimation and productivity assessment (Konior and Szóstak, 2020). The nature of an ERP implementation project results in the progress growing rapidly during the training and configuration phases, more slowly in the testing and conversion phases and reaching an asymptotic maximum when the project goes live (Fig. 1). The progress curve presented in Fig. 1 is a logistic curve, being similar to the S-Curve but ignoring the start-up effect in the project planning stage. The logistic model is noted for its robustness and is frequently used to predict and model the performance of an ERP project team (Plaza and Rohlf, 2008; Plaza et al., 2010), to measure project complexity (Dao et al., 2020), and to analyse productivity changes and financial implications of the introduction of new technology (Dardan et al., 2006). Plaza and Rohlf (2008) demonstrated that, for a project team working on a CSF, progress follows a logistic curve. This is also the case for teams working on other CSFs, vendor support, end users, IT infrastructure and top management (Sun et al., 2005). Inside an organisation, teams addressing such CSFs usually lack experience with, and knowledge of, the systems that they are implementing. Outwith the organisation, the ERP vendors' team are usually relatively ignorant about, and lack experience of, the client. For these reasons, the early impact on the ERP implementation performance of a particular CSF is low, improving with time to a peak threshold of performance at some point during the project.

We use the logistic curve in Fig. 1 to model the relationship between practice and performance. A critical predictor to measure the success of ERP implementation is claimed to be the system usage i.e. system's ability in performing tasks (Nwankpa and Roumani, 2014); it is claimed that the higher system usage by the end-user increases the chances of organisation's achieving ERP implementation objectives (Sun et al., 2005; Nwankpa, 2015). Xie et al. (2014) defined the performance level as the percentage of the organisation's target functional requirement met by the ERP implementation. In this research, practice is modelled as project duration *T*, while the overall ERP implementation performance level *PF* is measured as the estimated percentage of the company's functional requirements that are met by the ERP implementation. For example, the ERP system project manager estimated that only 50% of the company's functional requirements are met by the ERP implementation, which means PF=50.

When addressing individual CSFs, practice is represented by time spent on addressing ${\mathrm{CSF}}_{i}$, denoted by $t_{i}$, and performance means the progress achieved by ${\mathrm{CSF}}_{i}$, denoted by ${\mathrm{PF}}_{i}$ and measured as that CSF's percentage contribution to the overall ERP implementation performance level. A Progress vs Time logistic curve is used to express the relationship between the progress made by a project team against time to address a ${\mathrm{CSF}}_{i}$ and is formulated in (1):

(1) $${\mathrm{PF}}_{i}(t_{i})=p_{i}\cdot (1-e^{-k_{i}t_{i}})$$

where $p_{i}$ is the performance threshold, or the maximum percentage contribution a CSF makes to the overall performance level. The progressing coefficient $k_{i}$ relates directly to the rate of progress made by a team; however, since the ERP project team is diverse by nature, and will vary considerably with the context within which ERP is implemented (Yoon, 2009), $k_{i}$ is difficult to calculate accurately. The overall performance level of ERP implementation is calculated as:

(2) $$\mathrm{PF}(t_{1},t_{2},\ldots ,t_{M})=\sum_{i=1}^{M}{\mathrm{PF}}_{i}(t_{i})$$

### 3.3 Modelling cost against time

The overall ERP implementation performance level relies on the performance contributed by the individual CSFs. Administration of any CSF will usually require money, management effort and human resources. As such, the overall ERP implementation cost is calculated as the total cost of improving the CSFs. Based on the authors' observations and research on ERP implementations (Sun et al., 2005), the overall ERP implementation cost increases with the total time spent. A linear cost function is therefore constructed for each CSF, showing the required money against time spent to address the corresponding factor.

Adopting a uniform function to model the Cost vs Time relationship for all CSFs is used to simplify the functional form and focus on optimisation. Although some costs may be incurred when no time is spent on addressing CSFs, those costs are so low relative to the costs incurred in spending time that they can effectively be regarded as zero (Xie et al., 2014). The constant initial cost when $t_{i}=0$ is often omitted in the cost functions that model relationships between project schedules and costs of ERP implementation, as evidenced in Sun et al. (2005) and Plaza and Rohlf (2008). Therefore, we assume that ${\mathrm{cost}}_{i}(t_{i})=0$ when $t_{i}=0$, and the functional model of the cost to address CSF *i* is:

(3) $$\text{Linear model }{\mathrm{cost}}_{i}(t_{i})=d_{i}\cdot t_{i},\;t_{i}>0$$

The implementation cost of ERP is obtained as:

(4) $$C(t_{1},t_{2},\ldots ,t_{M})=\sum_{i=1}^{M}{\mathrm{cost}}_{i}(t_{i})$$

### 3.4 Development of constrained nonlinear programming model for ERP implementation

A CNL_ERP model is constructed as a nonlinear programming model defined by an objective and a set of constraints in (5). The objective is to maximise the ERP implementation performance level, subject to limitations on budget and project duration:

(5) $$\text{Max}\;\mathrm{PF}(t_{1},t_{2},\ldots ,t_{M})=\sum_{i=1}^{M}{\mathrm{PF}}_{i}(t_{i})=\sum_{i=1}^{M}p_{i}\cdot \left(1-e^{-k_{i}t_{i}}\right)$$

$$\begin{array}{cc} \text{s.t.}\; & g_{1}(t_{1},t_{2},\ldots ,t_{M})=\sum_{i=1}^{M}t_{i}\leq TL\\ \; & g_{2}(t_{1},t_{2},\ldots ,t_{M})=\sum_{i=1}^{M}{\mathrm{cost}}_{i}(t_{i})\leq \mathrm{CL}\\ \; & t_{i}\geq 0\;\text{for all }i=1,2,\ldots M \end{array}$$

Define vector $T={\left(t_{1},t_{2},\ldots ,t_{M}\right)}^{T}$ and formula (5) can be rewritten in matrix notation:

(5.a) $$\text{Max}\;\mathrm{PF}(T)=\sum_{i=1}^{M}{\mathrm{PF}}_{i}(t_{i})=\sum_{i=1}^{M}p_{i}\cdot (1-e^{-k_{i}t_{i}})$$

$$\begin{array}{cc} \text{s.t.}\; & g_{1}(T)=\sum_{i=1}^{M}t_{i}\leq TL\\ \; & g_{2}(T)=\sum_{i=1}^{M}{\mathrm{cost}}_{i}(t_{i})\leq \mathrm{CL}\\ \; & T\geq 0 \end{array}$$

The constrained nonlinear programming model in (5) or (5.a) is not explicitly solvable for symbolic solutions, but, if parameter values are provided, can be solved numerically using optimisation tools such as Excel's Solver, Mathematica and CPlex. The optimisation tools implement different algorithms depending on which solver is used; we use the Generalised Reduced Gradient (GRG) method that is a generalisation of the Steepest Ascent (or Steepest Descent) method. GRG converts the constrained problem into an unconstrained one by using direct substitution and uses an iterative procedure to find an improved direction for the objective function while satisfying the constraint equations; the improved direction is determined by the reduced gradient. Discussion of algorithms for nonlinear programming is beyond the scope of this research, but the GRG method is to be used to analyse the model in Section 3.4 and obtain implementation strategy in Section 4.4.

### 3.5 Analysis of the constrained nonlinear programming model

The reduced gradient of objective function PF(T) with respect to **T** is derived as $\nabla_{r}PF(T)$ in (6) (see Appendix A):

(6) $$\nabla_{r}\mathrm{PF}(T)=(\frac{\partial \mathrm{PF}}{\partial t_{1}},\frac{\partial \mathrm{PF}}{\partial t_{2}},\ldots ,\frac{\partial \mathrm{PF}}{\partial t_{M}})=(p_{1}k_{1}e^{-k_{1}t_{1}},p_{2}k_{2}e^{-k_{2}t_{2}},\ldots ,p_{M}k_{M}e^{-k_{M}t_{M}})$$

The extreme point $T^{⁎}={\left(t_{1}^{⁎},t_{2}^{⁎},\ldots ,t_{M}^{⁎}\right)}^{T}$ will be generated in the direction of the gradient in such a way that the maximum $\mathrm{PF}(T^{⁎})$ is achieved and constraints hold. It is obvious that the reduced gradient of $\nabla_{r}\mathrm{PF}(T)$ changes with **T**. The gradient in (6) not only varies with the $p_{i}$ and $k_{i}$, but also with time $t_{i}$ allocated to ${\mathrm{CSF}}_{i}$. A CSF with high performance threshold $p_{i}$, and fast learning or knowledge absorption speed which corresponds to a higher progressing coefficient $k_{i}$, contributes to the ERP implementation performance more quickly during the early part of the project duration. Time allocated to ${\mathrm{CSF}}_{i}$ in the next iteration is proportional to the value of $p_{i}k_{i}e^{-k_{i}t_{i}}$. The mathematical algorithm reveals that the focus given to CSFs should be adjusted according to the amount of resources available, usually involving time and budget, and the progressing coefficient $k_{i}$.

## 4 Empirical studies and results

A survey was conducted to collect empirical data including ERP project costs, schedules and performance levels from a group of SMEs. Statistical regression techniques were used to fit the empirical data to the analytical models for ERP cost and performance at CSF level, as shown in equations (1)–(4). Case studies were then conducted to check the validity and effectiveness of the analytical models.

### 4.1 Survey results

The initial questionnaire was developed based on the five CSFs identified in Table 1, focusing on the progress, cost and time associated with each CSF during implementation. The one-off costs of software and hardware are not included in the cost to address IT infrastructure. Based on the criteria in Table 2, we used Thomson Data, small business association websites, SAP user groups and ERP suppliers' websites to choose 400 SMEs and conducted internet-based surveys with them. 60 valid responses were received, giving a 15% response rate (see Appendix B). Those 60 SMEs cover a wide range of industrial sectors, including Manufacturing (28%), Information Technology (15%), Telecommunications (14%), Banking and Finance (10%), Utilities (9%), Education (2%), and others (23%). This ensures the sample is representative and the regression model is relatively more generalisable.

**Table 2:** Sampling criteria used in the survey.

**Location**	UK or North America
**Size**	50-150 employees
**ERP implementation**	Completion of at least one ERP project
**CSFs**	All the five CSFs are addressed during the ERP implementation

### 4.2 Analytical regression models for the observed data

Employing formulae (1) and (3), the values of $d_{i}$, $k_{i}$, $p_{i}$ and $R^{2}$ were obtained using least square methods for each CSF and presented in Table 3. $R^{2}$ values for both the Linear Cost vs Time curve and the Logistic Progress vs Time curve are above 0.70, indicating a good level of fit.

**Table 3:** Coefficients and ***R***^**2**^ of Cost and Progress regression models.

Parameters		***CSF***_**1**_-TM	***CSF***_**2**_-Users	***CSF***_**3**_-PM	***CSF***_**4**_-IT	***CSF***_**5**_-VS	Average ***R***^**2**^
Linear Cost model	*d*_*i*_	659.92	656.28	719.66	1361	1770.7	0.75
	$R_{i}^{2}$	0.91	0.61	0.68	0.77	0.79	
Logistic Progress model	*k*_*i*_	0.045	0.163	0.040	0.076	0.143	0.77
	*p*_*i*_	19.03	17.13	24.26	19.28	12.94	
	$R_{i}^{2}$	0.98	0.61	0.83	0.77	0.66	

For each CSF, the Cost vs Time linear curve and Progress vs Time exponential curve are formulated in equations (7)–(16):

(7) $${\mathrm{CSF}}_{1}\text{-TM:}\;{\mathrm{cost}}_{1}(t_{1})=659.92\cdot t_{1}$$

(8) CSF1-TM:PF1(t1)=19.03⋅(1−e−0.045⋅t1)

(9) $${\mathrm{CSF}}_{2}\text{-Users:}\;{\mathrm{cost}}_{2}(t_{2})=656.28\cdot t_{2}$$

(10) CSF2-Users:PF2(t2)=17.13⋅(1−e−0.163⋅t2)

(11) $${\mathrm{CSF}}_{3}\text{-PM:}\;{\mathrm{cost}}_{3}(t_{3})=719.66\cdot t_{3}$$

(12) CSF3-PM:PF3(t3)=24.26⋅(1−e−0.04⋅t3)

(13) $${\mathrm{CSF}}_{4}\text{-IT:}\;{\mathrm{cost}}_{4}(t_{4})=1361\cdot t_{4}$$

(14) CSF4-IT:PF4(t4)=19.28⋅(1−e−0.076⋅t4)

(15) $${\mathrm{CSF}}_{5}\text{-VS:}\;{\mathrm{cost}}_{5}(t_{5})=1770.7\cdot t_{5}$$

(16) CSF5-VS:PF5(t5)=12.94⋅(1−e−0.143⋅t5)

### 4.3 Case studies

#### 4.3.1 Details of ERP implementation projects

To demonstrate the practical benefits of the CNL_ERP model, and evaluate its effectiveness, we collected data from three real ERP implementations and applied CNL_ERP to them. The case study selection criteria are:

1) The selected organisations are SMEs who meet our sampling criteria in Table 2 and who participated in the survey.
2) The selected organisations implemented ERP systems on a similar schedule, for example within 110 days or 120 days, or implemented them at similar costs, for example at a cost of $100,000. This is to enable direct comparisons of resource allocation and resultant ERP performance between the organisations.

The methodology presented in this section can be used by a project manager to plan, prepare and deploy resources, and select training and implementation strategies. Two of the authors were participant observers on projects 1 and 3 respectively and the research team conducted post-implementation data collection on all three projects. CNL_ERP is initially tested and evaluated using real data to demonstrate the practical benefits of the model and to identify areas for improvement; testing with multiple case study companies generates more robust and precise results.

Observed data from each case are set up as a Baseline model, and the case analysis involves two test scenarios as follows:

**Scenario 1:** obtaining the project outcomes by inputting the observed $T={\left(t_{1},t_{2},\ldots ,t_{5}\right)}^{T}$ to the CNL_ERP, and releasing the constraints on planned project duration and budget;
**Scenario 2:** calculating ideal solution $T^{⁎}={\left(t_{1}^{⁎},t_{2}^{⁎},\ldots ,t_{5}^{⁎}\right)}^{T}$, and associated project outcomes by maximising the project performance level whilst satisfying constraints on planned project duration and budget.

Scenario 1 is used to test the validity and effectiveness of the CNL_ERP, checking as to whether the outputs from CNL_ERP are similar to the observed results if the inputs $T{\left(t_{1},t_{2},\ldots ,t_{5}\right)}^{T}$, i.e. resources assigned to each CSF, are the same. Scenario 2 aims to compare resource allocations and associated ERP results from real life situations against those recommended by CNL_ERP. Applying CNL_ERP to the case studies offers an opportunity to conduct preliminary tests and to identify the differences between the resources allocated by SMEs in real life situations and resources assigned by CNL_ERP, as well as the differences between the resulting project outcomes. The case studies also evaluate the analytical and practical aspects of CNL_ERP as a tool for predicting and allocating resources prior to implementation.

Due to confidentiality agreements and privacy, we refer to the case companies anonymously as Company 1, 2 and 3. Company 1 is a US based company designing and manufacturing switches, routers and other networking equipment for clients in the governmental, corporate and educational sectors. They implemented three modules of SAP ERP within a planned budget of $100,000, including Financial Accounting, Materials Management and Production Planning. Company 2 is based in the UK and is a supplier of booking and membership systems for universities, leisure centres, clubs and health and fitness groups. The company implemented two SAP ERP modules in four months, namely Sales and Financial Accounting. Company 3 is also UK based and concentrates on supplying management software packages to schools, colleges and universities; they implemented a heavily customised version of Priority ERP software, with the core being the Sales and Financial Accounting modules. Detailed information about the companies and their ERP implementations is shown in Table 4.

**Table 4:** Information about case companies.

	Case Company 1	Case Company 2	Case Company 3
Participant's Job Title	MIS-Manager	SQA-Analyst	Net-Developer
Industry	IT	Leisure Industry	Education
Location	USA	UK	UK
No. of employees	118	220	240
No. of internal resources + external consultants	3 + 5	5 + 1	4 + 2
Implementation result	Successful	Successful	Successful
Implementation completed on time?	Yes	Yes	Yes
Completed within budget?	Yes	Yes	Yes
Project duration (Days): *T*	120	107	110
Cost of implementation: *C*	$100,000	$90,000	$90,000
Project Performance Level *PF*	65%	70%	63%

The following information was collected during interviews with the participants from Case Companies 1-3, and recorded in the “Baseline” row in Table 5: (1) $t_{i}$ - time spent on the ${\mathrm{CSF}}_{i}$, (2) *T* - project duration, (2) *C* - implementation cost, (3) *PF* – performance level.

**Table 5:** Comparison of observed results and results generated by CNL_ERP.

Case	Test	*t*_1_	*t*_2_	*t*_3_	*t*_4_	*t*_5_	*T*	*C*	*PF*	*PI*	Cost difference	Performance difference
1	Baseline	30	40	30	10	10	120	$100,000	65%	0.69	0	0
	Scenario 1	30	40	30	10	10	120	$100,000	68%	0.69	0	+3%
	Scenario 2	33	16	39	17	9	114	$100,000	73%	1	0	+8%

2	Baseline	28	28	21	15	15	107	$90,000	70%	0.71	0	0
	Scenario 1	28	28	21	15	15	107	$98,941	69%	0.71	+$8,941	−1%
	Scenario 2	30	15	35	15	8	103	$90,000	70%	1	0	0

3	Baseline	20	40	20	10	20	110	$90,000	63%	0.62	0	0
	Scenario 1	20	40	20	10	20	110	$102,866	64%	0.62	+$12,866	+1%
	Scenario 2	30	15	35	15	8	103	$90,000	70%	1	0	+7%

By setting up a goal of maximising the ERP performance level, CNL_ERP was applied to make decisions on $T={\left(t_{1},t_{2},\ldots ,t_{5}\right)}^{T}$ for the Case Companies 1-3. The objective function is formulated in equation (17) for the three case studies. Substituting (8), (10), (12), (14) and (16) to (17), the objective function becomes:

(17) $$\text{Max}\;\mathrm{PF}(T)=19.03\cdot (1-e^{-0.045\cdot t_{1}})+17.13\cdot (1-e^{-0.163\cdot t_{2}})+24.26\cdot (1-e^{-0.04\cdot t_{3}})+19.28\cdot (1-e^{-0.076\cdot t_{4}})+12.94\cdot (1-e^{-0.143\cdot t_{5}})$$

Subject to different constraints in (18) and (19):

(18) $$\begin{array}{ccc} \text{Case 1:}\; & \text{s.t.}\; & g_{1}(T)=\sum_{i=1}^{5}t_{i}\leq 120\\ \; & \; & g_{2}(T)=\sum_{i=1}^{5}{\mathrm{cost}}_{i}(t_{i})\leq 100,000\\ \; & \; & T\geq 0 \end{array}$$

(19) $$\begin{array}{ccc} \text{Case 2 and Case 3:}\; & \text{s.t.}\; & g_{1}(T)=\sum_{i=1}^{5}t_{i}\leq 120\\ \; & \; & g_{2}(T)=\sum_{i=1}^{5}{\mathrm{cost}}_{i}(t_{i})\leq 90,000\\ \; & \; & T\geq 0 \end{array}$$

#### 4.3.2 Discussion of results

**Scenario 1:** As shown in Table 5, the observed $t_{i}$ from **Scenario 1** is used as an input to Equation (17); the Performance level *PF* achieved in **Scenario 1** is 68% for Case 1, 69% for Case 2, and 64% for Case 3, similar to the observed *PF* in the Baseline model, i.e. 65% for Case 1, 70% for Case 2, and 63% for Case 3. Inputting observed data to CNL_ERP generates a similar implementation cost as the observed cost. The results from the case studies verify the validity and accuracy of the CNL_ERP model, showing that the integrated model closely resembles the actual ERP implementation performances. The small difference in costs could be explained as being due to the constructed analytical Cost vs Time model being obtained through the regression fitness approach and, therefore, not producing the same results as the observations.
**Scenario 2:** Equation (17) is solved by Mathematica using the GRG procedure. The calculated $T^{⁎}={\left(t_{1}^{⁎},t_{2}^{⁎},\ldots ,t_{5}^{⁎}\right)}^{T}$, implementation cost *C* and ERP performance level *PF* in **Scenario 2** are compared with the observed results in the Baseline model in Table 5. Given **T** is a vector, we define a Proximity Index *PI* to compare the similarity of the observed $T={\left(t_{1},t_{2},\ldots ,t_{5}\right)}^{T}$ to the calculated $T^{⁎}={\left(t_{1}^{⁎},t_{2}^{⁎},\ldots ,t_{5}^{⁎}\right)}^{T}$. We adopt the Similarity to Ideal Solution proposed by Hwang and Yoon (1981) to calculate *PI* using the Technique of Order Preference by Similarity to Ideal Solution (TOPSIS), as shown in (20):

(20) $$\mathrm{PI}=S^{-}/(S^{+}+S^{-})$$

where

(21) $$S^{+}=\sqrt{(T-T^{⁎}){\left(T-T^{⁎}\right)}^{T}}$$

and

(22) $$S^{-}=\sqrt{(T-T^{-}){\left(T-T^{-}\right)}^{T}}$$

In equations (21) and (22), $T^{⁎}$ is the extreme point and used as the positive ideal solution, and $T^{-}=(0,0,0,0,0)$ is the negative ideal solution which minimises the performance level *PF* to be 0. $S^{+}$ and $S^{-}$ are the separations from positive and negative ideal solutions, respectively. The higher the proximity *PI*, the closer the observed $T={\left(t_{1},t_{2},\ldots ,t_{5}\right)}^{T}$ to the calculated $T^{⁎}={\left(t_{1}^{⁎},t_{2}^{⁎},\ldots ,t_{5}^{⁎}\right)}^{T}$.

In Case 1, the observed $T={\left(30,\;40,\;30,\;10,\;10\right)}^{T}$ is very different from the calculated $T^{⁎}={\left(33,\;16,\;39,\;17,\;9\right)}^{T}$, with the proximity indicator *PI* being 0.69. PF=65% in the Baseline, is lower than the PF=73% achieved in Scenario 2. In the observed data, Case 1 allocated more time (40 days) to Users than to Top Management (30 days) as Users are more compliant and have more time to spend on implementation. It is also less expensive to address Users as a CSF. According to CNL_ERP, more time should be allocated to Top Management (33 days) than to Users (16 days). The MIS Manager in Case 1 explained that ‘… *in SMEs, senior managers do not usually have experience of implementing ERP projects and also have many other responsibilities*. …. *In an SME, ERP implementation is a choice; to implement it, your manager has to be convinced of the benefit. The manager did not have great interest in ERP implementation, and he does not have time for it either* … *while users are more willing to spend time learning the new software. The cost of having more users in the project is much cheaper than having the upper management team involved*.” Although a strong commitment from Top Management would be extremely valuable to the ERP implementation, Top Management in many SMEs often cannot afford this kind of commitment, and instead would involve alternative resources such as Users. This conclusion is consistent with the conclusion drawn from Plaza and Rohlf (2008).

Cases 2 and 3 have the same amount of resources in terms of time and budget available, therefore the calculated solutions are identical as $T^{⁎}={\left(30,\;15,\;35,\;15,\;8\right)}^{T}$. Comparing the results from the two cases, we observed that:

1) The observed $T={\left(28,\;28,\;21,\;15,\;15\right)}^{T}$ in Case 2 is more similar to the $T^{⁎}={\left(30,\;15,\;35,\;15,\;8\right)}^{T}$ recommended by CNL_ERP, with the PI=0.71. Time allocation in Case 1, $T={\left(20,\;40,\;20,\;10,\;20\right)}^{T}$, is more distant from $T^{⁎}={\left(30,\;15,\;35,\;15,\;8\right)}^{T}$, having the PI=0.62.
2) As a result of the closeness to the ideal solution, a higher performance level is achieved in Case 2 with PF=70% as opposed to PF=63% obtained in Case 3.
3) The results presented in Table 5 confirm that allocating time as recommended by CNL_ERP produces higher performance levels while incurring lower costs. It should be noted that, generally, focusing efforts and resources on CSFs as recommended by CNL_ERP leads to better outcomes.

However, the interview results from Case 1 indicate that the most effective approach is to combine expert judgement with the CNL_ERP recommendation and make appropriate adjustments to the CNL_ERP recommendation wherever appropriate.

### 4.4 ERP implementation strategy

Once resources are allocated to CSFs prior to ERP implementation, the next step is to develop an implementation strategy realising resource allocation to CSFs by finding out if CSFs should be addressed sequentially or simultaneously, or a combination of both. SMEs tend to focus on one CSF and finish it before moving to the next; however, CSFs are inter-related and should be considered in all phases of ERP implementation (Sun et al., 2005). CNL_ERP can provide guidance in developing implementation strategies by ensuring the pre-determined performance level is achieved under budget and time limits.

**Figure 2:**
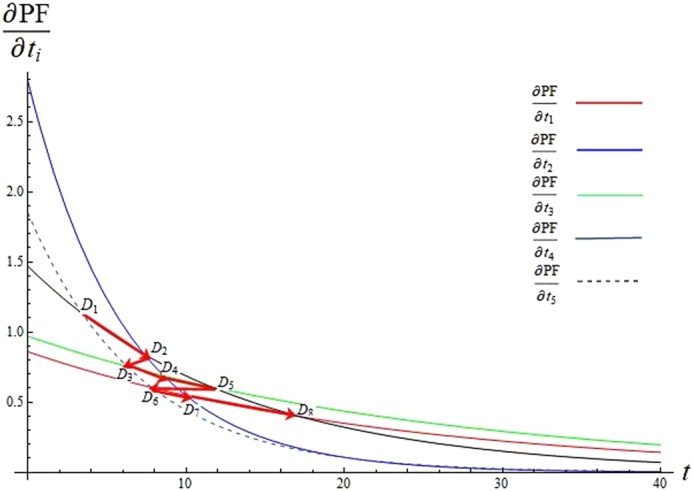
Dimensional elements $\frac{\partial \mathrm{PF}}{\partial t_{i}}$ in reduced gradient ∇_*r*_*PF*.

**Figure 3:**
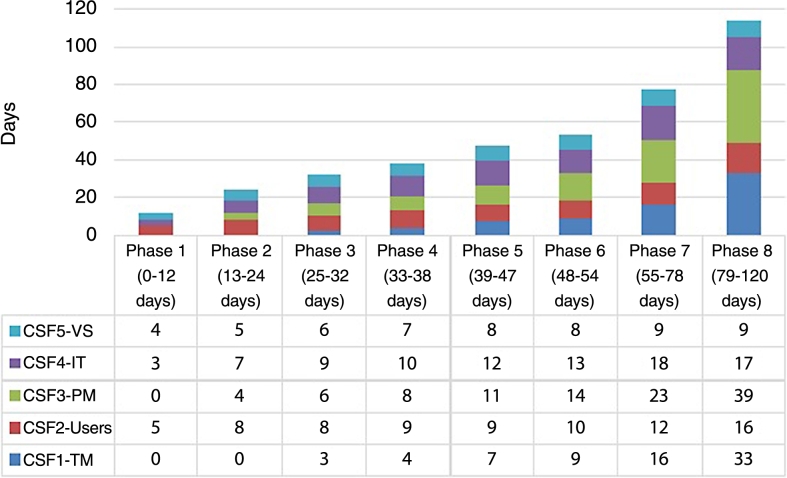
ERP implementation strategy developed by CNL_ERP.

Using Case 1 as an example, an implementation strategy can be developed by maximising the performance level in different time periods while the budget is controlled to be under $100,000. Substituting values of $p_{i}$ and $k_{i}$ to (6), the reduced gradient of *PF* is given in equation (23), and dimensional elements $\frac{\partial \mathrm{PF}}{\partial t_{i}}$ are plotted in Fig. 2. From a mathematical point of view, points $D_{n}(t_{i},\frac{\partial \mathrm{PF}}{\partial t_{i}})$ (n=1,2…,8) in Fig. 2 are where $\frac{\partial \mathrm{PF}}{\partial t_{i}}$ intersect, and the red arrows indicate how the search direction for $T^{⁎}$ changes when *TT* increases; while in practice, points $D_{i}$ break the implementation period into 8 phases ($D_{5}$ occurs almost at the same time as $D_{6}$ when TT=47) and resource is allocated to ${\mathrm{CSF}}_{i}$ based on their priorities which are determined by $\frac{\partial \mathrm{PF}}{\partial t_{i}}$ and corresponding constraints. The numerical values of $D_{i}(t_{i},\frac{\partial \mathrm{PF}}{\partial t_{i}})$ and associated *TT* are listed in Table 6, based on which an implementation strategy is developed in 8 phases and resources are allocated to CSFs according to their priorities (see Fig. 3). The priorities of CSFs do not stay the same but change along with the constraints imposed at each phase.

(23) $$\nabla_{r}\mathrm{PF}(T)\;=(\frac{\partial \mathrm{PF}}{\partial t_{1}},\frac{\partial \mathrm{PF}}{\partial t_{2}},\ldots ,\frac{\partial \mathrm{PF}}{\partial t_{5}})\;=(0.86e^{-0.045t_{1}},2.79e^{-0.163t_{2}},0.97e^{-0.04t_{2}},1.47e^{-0.076t_{2}},1.85e^{-0.143t_{5}})$$

**Table 6:** Numerical solutions of *D*_*i*_ and associated *TT*.

*D*_*n*_	*D*_1_	*D*_2_	*D*_3_	*D*_4_	*D*_5_	*D*_6_	*D*_7_	*D*_8_
$D_{n}(t_{i},\frac{\partial PF}{\partial t_{i}})$	(3.43, 1.13)	(7.37, 0.84)	(6.27, 0.75)	(8.59, 0.69)	(11.55, 0.61)	(7.82, 0.60)	(9.97, 0.60)	(17.29, 0.39)
*TT* (days)	12	24	32	38	47	48	54	78

Since project planning is assumed to be complete, an implementation strategy can be obtained as follows when the budget limit is $100,000 and time limit is 120 days: in Phase 1 (0∼12 days), once agreement and support have been obtained from TM, and the project team has been formed up in the planning phase, TM and PM do not need to make much commitment to the project, while training should be provided to Users and data migration should be started with IT and VS; in Phase 2 (13-24 days), PM will start while the main focus is still on Users, IT and VS; in Phase 3 (25-32 days), TM starts making commitment, for example, checking project progress and providing necessary support; in Phases 4, 5, and 6 (33-54 days), PM takes over VS, Users, and IT respectively, and should be allocated more time; once Users are trained and data migration is nearly done, PM should be in charge of the whole project and monitor its progress until success occurs; finally, in Phases 7 and 8 (55-115 days), TM takes over Users and IT and becomes the second most important CSF, which should be addressed with an increased time resource in order to ensure that the implementation is successful and within budget.

The strategy for implementing ERP was developed from mathematical analysis of ERP implementation performance, and the MIS manager in Case 1 explained that a real project may not be able to follow each phase as suggested here as each project is situational and has different characteristics. However, he advised that the plan obtained here does provide a rich picture of where to concentrate effort in the initial, intermediate and final phases, and is very helpful in enabling an SME to understand the progress of an ERP project and the resources needed, and therefore make appropriate arrangements or preparations. For example, in initial phases 1 and 2, full time staff can be released from PM and TM teams while VS and Users need to be in place; in intermediate phases 3 and 4, some but not all staff from PM and TM need to work on the project; and in final phases 5 to 8, demands on PM and TM become more intensive and staff accountable should be reserved for this period, while VS will not be needed and staff from IT and Users training can be reduced.

### 4.5 Impacts of progressing coefficient $k_{i}$

The case studies in Section 4.3 provide helpful guidance when allocating resources prior to ERP implementation. However, if extra resource, such as budget, becomes available to provide ERP training, which CSF (or the team that addresses the CSF) shall we make investment in? Training increases the progress coefficient $k_{i}$ which is the critical parameter affecting the overall project duration and implementation performance. A change $\Delta k_{i}$ will cause change $\Delta {\mathrm{PF}}_{i}$, as calculated in (24)-(26):

(24) $$\Delta {\mathrm{PF}}_{i}={\mathrm{PF}}_{i}(k_{i}+\Delta k_{i})-{\mathrm{PF}}_{i}(k_{i})$$

Substituting (1) to (24),

(25) $$\Delta {\mathrm{PF}}_{i}=p_{i}\cdot e^{-k_{i}t_{i}}\cdot (1-e^{-\Delta k_{i}t_{i}})$$

The maximal $\Delta {\mathrm{PF}}_{i}$ is obtained when $\Delta k_{i}\to \infty$, hence

(26) $$\lim_{\Delta k_{i}\to \infty }\Delta {\mathrm{PF}}_{i}=p_{i}\cdot e^{-k_{i}t_{i}}$$

**Figure 4:**
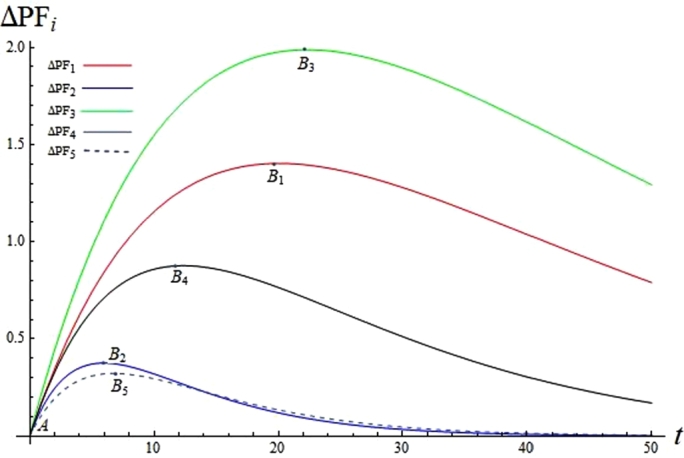
Changes of CSF's progress Δ*PF*_*i*_ as a function of *t*_*i*_, when Δ*k*_*i*_ = 0.01.

The progressing coefficient $k_{i}$ is directly related to the rate of performance improvement. Changing $k_{i}$ causes a shift in the CSF's progress level, noted as $\Delta {\mathrm{PF}}_{i}$, which leads to a shift in the overall ERP implementation performance level. Applying (25) to Case 1, in Fig. 4 we depict the impacts of progressing coefficient variation ($\Delta k_{i}=0.01$) on a CSF's progress level change ($\Delta {\mathrm{PF}}_{i}$) as a function of $t_{i}$. Fig. 4 shows that an identical change in the progressing coefficient ($\Delta k_{i}=0.01$) has a different impact on different CSFs, with the order of impact being, from strongest to weakest: ${\mathrm{CSF}}_{3}$ (PM), ${\mathrm{CSF}}_{1}\;(\mathrm{TM}),\;{\mathrm{CSF}}_{4}$ (IT), ${\mathrm{CSF}}_{2}$ (Users) and ${\mathrm{CSF}}_{5}$ (VS). At the same time point $t_{i}$ and with the same level $\Delta k_{i}$, the ${\mathrm{CSF}}_{i}$ with the higher $p_{i}e^{-k_{i}t_{i}}$ achieves a higher $\Delta {\mathrm{PF}}_{i}$. As shown in Fig. 4 and Table 7, when $\Delta k_{i}=0.01$, the maximum $\Delta {\mathrm{PF}}_{i}$ is achieved at the point $B_{i}(t_{i},\;\Delta {\mathrm{PF}}_{i}$). Among all the CSFs, ${\mathrm{CSF}}_{3}$ achieves the highest value of $B_{3}=1.99$, while ${\mathrm{CSF}}_{5}$ obtains the lowest $B_{5}=0.32$. The impact of the progressing speed increment $\Delta k_{5}$ on ${\mathrm{CSF}}_{5}$’s progress level is less significant in comparison with other CSFs. This shows that training provided to CSFs that have low potential (i.e., low $p_{i}e^{-k_{i}t_{i}}$) would be unproductive in terms of improving the progress level or the overall ERP implementation performance level. The results presented in Fig. 4 and Table 7 confirm that the most cost-effective strategy is to offer training to CSFs that have a high potential in making contributions to the overall implementation performance, i.e. ${\mathrm{CSF}}_{3}$.

**Table 7:** Impacts of *k*_*i*_ on *CSF*_*i*_'s progress *PF*_*i*_.

	T^⁎^ = (33,16,39,17,9) Δ*k*_*i*_ = 0.01	$\Delta k_{i}=0.01$	
Δ*PF*_1_	1.21	*B*_1_	(20, 1.40*)
Δ*PF*_2_	0.19	*B*_2_	(6, 0.38*)
Δ*PF*_3_	1.65	*B*_3_	(22, 1.99*)
Δ*PF*_4_	0.83	*B*_4_	(12, 0.88*)
Δ*PF*_5_	0.31	*B*_5_	(7, 0.32*)
Δ*PF*	4.19		

* when $\Delta k_{i}=0.01$, the maximal $\Delta {\mathrm{PF}}_{i}$ is achieved at point $B_{i}$

We calculate the values of $\Delta {\mathrm{PF}}_{i}$ when $\Delta k_{i}=0.01$ at the extreme point $T^{⁎}=(33,16,39,17,9)$, as shown in the second column in Table 7. Compared with other CSFs, ${\mathrm{CSF}}_{3}$ (PM) and ${\mathrm{CSF}}_{1}\;(\mathrm{TM})$ are more sensitive to $\Delta k_{i}$. Increasing the progressing speed by $\Delta k_{i}=0.01$ for every CSF leads to an increment in the overall performance level by ΔPF=4.19. As would be expected, the amount of resource and effort required to increase the CSF progressing speed by $\Delta k_{i}=0.01$ varies from case to case. SMEs could use CNL_ERP as a tool to predict the expected increment in the overall implementation performance level, and to identify where to invest in CSFs to increase processing speed. CNL_ERP can also be used to judge if the required investment could be offset by the performance improvement, and to facilitate decision making.

## 5 Conclusions

### 5.1 Theoretical contributions

Notwithstanding the fact that much of the ERP literature has identified CSFs and their association to the implementation of ERP systems, there is a lack of quantitative measurement of CSFs' contributions to overall ERP implementation performance. Such a research gap results in a fragmentary understanding of how much and when to invest in CSFs to achieve target ERP implementation performance. Specific contributions from this paper to the ERP literature are: 1) quantitatively associating CSFs with ERP implementation and assessing the performance contributed by each CSF; 2) offering a useful analytical tool to accurately monitor progress made, and cost incurred, by each of the CSFs against time; 3) empirically testing and validating the effectiveness of investment in CSFs on ERP project success; and 4) demonstrating the potential of a nonlinear programming model as a method for planning, acquiring and deploying resources in IT implementation.

Furthermore, a specific contribution made to the resource allocation literature is that this research provides a solid theoretical basis for studying CSF based resource allocations in projects to implement ERP systems.

### 5.2 Managerial contributions

SMEs are significant actors in generating global economic growth. The advent of Cloud-based ERP services offers them greater opportunities to streamline their business activities through ERP implementation. In a similar manner to on-premises ERP solutions, Cloud-based ERP implementation requires adequate planning, resource acquisition and resource deployment to prepare an SME for the project. The quantitative model developed in this study, i.e., CNL_ERP, assesses CSFs' contributions to ERP implementation performance using empirical data collected from sample SMEs. Hence, it could be used in the pre-implementation stage to support SMEs with similar characteristics to determine the priorities of CSFs and acquire necessary resources; it could facilitate decision making in SMEs about how much and when to invest in each CSF in the implementation stage. CNL_ERP can also be used as a tool to accurately measure the progress made and the cost incurred for each CSF, and the progress of the overall ERP implementation. Furthermore, as CNL_ERP explicitly incorporates several managerial decisions, it can be used as a tool to fine-tune the model's behaviour, such as reaching maximum performance level targets set by management while satisfying resource constraints.

This research also uses the generalised reduced gradient approach to help SMEs develop appropriate ERP implementation strategy, with a focus on resource allocation/planning decision at different stages where resources vary.

### 5.3 Limitations and future work

This paper presents an innovative quantitative model for ERP implementation, namely CNL_ERP. Empirical studies show that CNL_ERP closely resembles the actual ERP implementation performances and that focusing efforts and resources on CSFs as recommended by CNL_ERP leads to better outcomes. Priorities of CSFs vary according to the resources (budget limitations) available, so focus on CSFs needs to be adjusted accordingly. CNL_ERP works by giving preference to the lower cost and faster progressing CSFs when the budget is low and shifting preference to the medium cost and slower progressing factors, which have the potential to achieve a high asymptotic maximum progress level, when increased budget is available. CNL_ERP switches preferences between CSFs in order to achieve the highest performance level for the lowest cost.

We also conclude that in cases where TM are very committed to the ERP implementation project (investing time and budget), or Time and Budget are invested to PM (project team training, improving competence), the ERP implementation performance will be enhanced.

The results of this research are: 1) The cost of ERP implementation increases along the time horizon, whereas the performance level reaches a maximum and then stagnates; 2) Priorities of CSFs vary according to the resources available (budget limitations), so focus on CSFs needs to be adjusted accordingly. We also conclude that, in cases where TM are very committed to the ERP implementation project (investing time and budget) or Time and Budget are invested to PM (project team training, improving competence), the ERP implementation performance will be enhanced.

Our analysis offers managers direct insights into CSF based resources (time and budget) when planning allocations, but it could be difficult to generalise the quantitative model to SMEs who do not meet the sampling criteria in Table 2. Future research will expand the sampling pool to involve SMEs with diverse characteristics and refine the CNL_ERP model using extra data sets. Further data collection and study is required to examine how the CSFs' progressing coefficients are influenced by external consulting, staff training and staff allocation; CNL_ERP can identify which resources are required to reach the necessary progressing speeds, thus enhancing and enabling ERP project planning and the development of training plans.

## Declarations

### Author contribution statement

**Ying Xie:** Conceived and designed the experiments; Analyzed and interpreted the data; Contributed reagents, materials, analysis tools or data; Wrote the paper.

**Colin Allen:** Conceived and designed the experiments; Analyzed and interpreted the data; Wrote the paper.

**Mahmood Ali:** Performed the experiments; Contributed reagents, materials, analysis tools or data.

### Funding statement

This research did not receive any specific grant from funding agencies in the public, commercial, or not-for-profit sectors.

### Data availability statement

Data included in article/supp.material/referenced in article.

### Declaration of interests statement

The authors declare no conflict of interest.

### Additional information

No additional information is available for this paper.

## Figures and Tables

**Table:** 

Company	Criteria	CSF1-TM	CSF2-Users	CSF3-PM	CSF4-IT	CSF5-VS	Total	Company	Criteria	CSF1-TM	CSF2-Users	CSF3-PM	CSF4-IT	CSF5-VS	Total
1	Time (days)	30	40	30	10	10	120	31	Time (days)	7	28	21	14	14	84
	Cost ($)	11,000	22,000	37,500	19,500	10,000	$100,000		Cost ($)	1,680	5,600	16,800	14,000	17,920	$56,000
	Progress (%)	7	15	10	11	7	65		Progress (%)	3	15	13	13	13	55

2	Time (days)	1	4	3	3	3	14	32	Time (days)	10	20	30	20	20	100
	Cost ($)	0	5,250	3,000	3,750	3,750	$15,750		Cost ($)	2,130	10,650	17,750	21,300	19,170	$71,000
	Progress (%)	0	20	0	0	0	20		Progress (%)	3	9	11	6	7	35

3	Time (days)	18	9	36	108	9	180	33	Time (days)	10	21	28	15	6	80
	Cost ($)	1,500	1,500	3,000	15,000	1,500	$22,500		Cost ($)	3,950	14,220	18,960	16,590	16,590	$70,310
	Progress (%)	3	10	8	25	5	50		Progress (%)	7	27	23	16	9	80

4	Time (days)	20	5	40	30	5	100	34	Time (days)	14	25	35	50	26	150
	Cost ($)	4,125	16,500	24,750	24,750	12,375	$82,500		Cost ($)	4,050	27,000	33,750	54,000	16,200	$135,000
	Progress (%)	10	25	25	25	15	100		Progress (%)	5	20	10	15	10	60

5	Time (days)	10	50	20	20	20	120	35	Time (days)	10	20	25	20	5	80
	Cost ($)	5,000	10,000	10,000	12,500	12,500	$50,000		Cost ($)	10,000	20,000	25,000	20,000	5,000	$80,000
	Progress (%)	13	20	20	18	10	80		Progress (%)	10	20	25	20	5	80

6	Time (days)	8	20	16	30	16	90	36	Time (days)	5	12	18	10	5	50
	Cost ($)	5,880	39,200	13,720	78,400	58,800	$196,000		Cost ($)	3,150	6,300	22,050	18,900	12,600	$63,000
	Progress (%)	7	18	24	22	11	80.5		Progress (%)	1	6.5	5	5	2.5	20

7	Time (days)	30	30	30	60	30	180	37	Time (days)	14	28	30	24	14	110
	Cost ($)	45,000	45,000	60,000	60,000	45,000	$255,000		Cost ($)	12,600	25,200	35,000	51,800	15,400	$140,000
	Progress (%)	28	5	23	8	8	70		Progress (%)	3	23	23	21	12	80

8	Time (days)	30	60	90	90	30	300	38	Time (days)	0	14	20	10	6	50
	Cost ($)	11,250	33,750	45,000	101,250	33,750	$225,000		Cost ($)	0	10,000	10,000	17,500	12,500	$50,000
	Progress (%)	25	8	25	15	8	80		Progress (%)	0	0	0	0	0	0

9	Time (days)	30	130	60	100	40	360	39	Time (days)	10	17	25	27	11	90
	Cost ($)	65,000	65,000	65,000	65,000	65,000	$325,000		Cost ($)	4,000	12,000	17,600	28,000	18,400	$80,000
	Progress (%)	10	10	30	25	5	80		Progress (%)	5	30	18	28	10	90

10	Time (days)	18	18	18	18	18	90	40	Time (days)	14	35	42	56	33	180
	Cost ($)	6,000	6,000	6,000	6,000	6,000	$30,000		Cost ($)	16,000	40,000	56,000	68,000	2,000	$182,000
	Progress (%)	14	14	14	14	14	70		Progress (%)	3	13	10	10	5	40

11	Time (days)	20	20	30	40	10	120	41	Time (days)	0	28	30	35	27	120
	Cost ($)	2,500	10,000	12,500	17,500	7,500	$50,000		Cost ($)	0	11,250	15,000	30,000	18,750	$75,000
	Progress (%)	10	15	19	18	12	73		Progress (%)	0	25	25	25	15	90

12	Time (days)	20	40	20	10	20	110	42	Time (days)	14	40	49	63	44	210
	Cost ($)	4,750	32,000	23,000	16,000	14,250	$90,000		Cost ($)	9,000	60,000	84,000	111,000	36,000	$300,000
	Progress (%)	10	15	13	10	15	63		Progress (%)	4	16	15	11	15	60

13	Time (days)	30	60	70	70	40	270	43	Time (days)	5	12	15	18	10	60
	Cost ($)	9,800	20,000	50,400	100,800	100,000	$281,000		Cost ($)	2,400	6,000	10,800	14,000	6,800	$40,000
	Progress (%)	18	13	23	23	5	80		Progress (%)	2	8	8	7	7	30

14	Time (days)	2	20	10	50	18	100	44	Time (days)	9	21	19	11	10	70
	Cost ($)	30,000	6,000	12,000	6,000	6,000	$60,000		Cost ($)	7,000	29,400	28,000	42,000	33,600	$140,000
	Progress (%)	0	10	5	35	10	60		Progress (%)	5	8	9	10	9	40

**Table:** 

Company	Criteria	CSF1-TM	CSF2-Users	CSF3-PM	CSF4-IT	CSF5-VS	Total	Company	Criteria	CSF1-TM	CSF2-Users	CSF3-PM	CSF4-IT	CSF5-VS	Total
15	Time (days)	10	25	30	35	20	120	45	Time (days)	7	21	21	21	14	84
	Cost ($)	8,000	16,000	20,000	24,000	12,000	$80,000		Cost ($)	2,920	11,680	14,600	25,550	18,250	$73,000
	Progress (%)	14	13	23	13	17	80		Progress (%)	10	36	20	22	12	100

16	Time (days)	45	30	180	180	30	465	46	Time (days)	10	21	28	37	19	115
	Cost ($)	40,000	80,000	100,000	80,000	20,000	$320,000		Cost ($)	3,300	51,150	39,600	54,450	16,500	$165,000
	Progress (%)	10	20	25	20	5	80		Progress (%)	6	15	15	14	11	60

17	Time (days)	14	56	21	70	21	182	47	Time (days)	7	21	28	21	13	90
	Cost ($)	15,000	75,000	50,000	175,000	85,000	$400,000		Cost ($)	1,350	24,300	39,150	40,500	29,700	$135,000
	Progress (%)	7.5	10	10	13	10	50		Progress (%)	0	10	7	7	7	31

18	Time (days)	28	28	21	15	15	107	48	Time (days)	0	14	20	10	6	50
	Cost ($)	20,000	30,000	10,000	20,000	10,000	$90,000		Cost ($)	0	12,720	11,660	15,900	12,720	$53,000
	Progress (%)	15	15	15	15	10	70		Progress (%)	0	26	18	19	8	70

19	Time (days)	14	28	21	21	30	114	49	Time (days)	5	14	18	18	8	63
	Cost ($)	18,000	45,000	27,000	63,000	27,000	$180,000		Cost ($)	810	16,200	21,870	26,730	15,390	$81,000
	Progress (%)	15	20	10	18	8	70		Progress (%)	5	18	10	14	14	60

20	Time (days)	10	45	35	28	14	132	50	Time (days)	0	7	7	7	7	28
	Cost ($)	20,000	60,000	20,000	60,000	40,000	$200,000		Cost ($)	0	6,150	10,250	14,350	10,250	$41,000
	Progress (%)	15	25	10	13	8	70		Progress (%)	0	5	5	5	5	20

21	Time (days)	90	90	90	90	90	450	51	Time (days)	12	21	30	33	19	115
	Cost ($)	50,000	50,000	100,000	100,000	200,000	$500,000		Cost ($)	8,850	53,100	44,250	53,100	17,700	$177,000
	Progress (%)	21	15	21	17	17	90		Progress (%)	8	25	20	13	15	80

22	Time (days)	14	76	28	21	21	160	52	Time (days)	0	12	20	12	11	55
	Cost ($)	9,000	81,000	24,000	150,000	36,000	$300,000		Cost ($)	0	11,850	15,800	31,600	19,750	$79,000
	Progress (%)	21	18	19	17	17	90.75		Progress (%)	0	7	8	7	9	30

23	Time (days)	21	52	28	28	28	157	53	Time (days)	7	28	18	21	6	80
	Cost ($)	11,250	45,000	13,500	123,750	34,875	$228,375		Cost ($)	2,760	15,640	23,000	27,600	23,000	$92,000
	Progress (%)	21	21	21	20	17	100		Progress (%)	13	24	19	18	18	90

24	Time (days)	7	35	14	14	14	84	54	Time (days)	7	21	21	28	13	90
	Cost ($)	2,550	29,700	6,800	40,000	5,950	$85,000		Cost ($)	0	10,710	15,750	22,050	14,490	$63,000
	Progress (%)	19	18	20	18	15	90		Progress (%)	0	6	4	4	6	20

25	Time (days)	4	4	10	4	6	28	55	Time (days)	14	35	35	60	41	185
	Cost ($)	800	6,000	6,000	5,200	2,000	$20,000		Cost ($)	7600	32300	32300	66500	51300	$190,000
	Progress (%)	0	3	3	3	3	10		Progress (%)	5	20	15	20	17.5	77.5

26	Time (days)	7	21	18	20	14	80	56	Time (days)	12	32	35	42	24	145
	Cost ($)	3,500	14,700	12,600	14,000	9,800	$54,600		Cost ($)	2,340	23,400	28,080	40,950	22,230	$117,000
	Progress (%)	10	28	23	15	15	90		Progress (%)	7.5	18	20.5	20	14	80

27	Time (days)	0	30	35	30	25	120	57	Time (days)	4	17	14	12	9	56
	Cost ($)	0	58,500	39,000	78,000	19,500	$195,000		Cost ($)	1,020	6,120	10,200	17,850	15,810	$51,000
	Progress (%)	0	33	25	20	13	90.5		Progress (%)	3	8	7	7	5	30

**Table:** 

Company	Criteria	CSF1-TM	CSF2-Users	CSF3-PM	CSF4-IT	CSF5-VS	Total	Company	Criteria	CSF1-TM	CSF2-Users	CSF3-PM	CSF4-IT	CSF5-VS	Total
28	Time (days)	8	23	17	35	20	103	58	Time (days)	0	14	21	35	15	85
	Cost ($)	3,000	36,250	24,650	50,750	30,450	$145,100		Cost ($)	0	10,800	15,840	21,600	23,760	$72,000
	Progress (%)	4	17	14	11	5	50		Progress (%)	0	29	24	20	18	90

29	Time (days)	21	35	25	39	20	140	59	Time (days)	8	14	20	12	11	65
	Cost ($)	16,800	42,000	31,500	94,500	42,000	$226,800		Cost ($)	1,740	15,660	22,620	25,230	21,750	$87,000
	Progress (%)	3	21	14	29	14	80		Progress (%)	6	19	19	21	17	80

30	Time (days)	5	42	33	65	25	170	60	Time (days)	8	12	10	15	5	50
	Cost ($)	2,100	16,100	21,000	21,700	9,100	$70,000		Cost ($)	5,200	12,350	14,950	17,550	14,950	$65,000
	Progress (%)	7.5	32.5	19.5	22.5	8	90		Progress (%)	2	2	2	2	2	10

## Appendix A Generalised reduced gradient

Converting the inequality constraints $g_{1}$ and $g_{2}$ in (5.a) into equations by adding nonnegative slack variables $S_{1}$ and $S_{2}$, the nonlinear optimisation problem becomes:

(A.1) $$\text{Max}\;\mathrm{PF}(T)=\sum_{i=1}^{M}p_{i}\cdot (1-e^{-k_{i}t_{i}})$$

$$\begin{array}{cc} \text{s.t.}\; & g_{1}(T)=\sum_{i=1}^{M}t_{i}-\mathrm{TL}+S_{1}=0\\ \; & g_{2}(T)=\sum_{i=1}^{M}{\mathrm{cost}}_{i}(t_{i})-\mathrm{CL}+S_{2}=0\\ \; & T\geq 0 \end{array}$$

Define

T=(Y,Z)

Let

$$Y\text{ be basic variables, }Z\text{ be nonbasic variables, and }g={\left(g_{1},\;g_{2}\right)}^{T}$$

The first partial derivatives of function PF(T) with respect to **Y** and **Z** is:

(A.2) $$\partial \mathrm{PF}(Y,Z)=\nabla_{Y}\mathrm{PF}\partial Y+\nabla_{Z}\mathrm{PF}\partial Z$$

The first partial derivatives of functions $g_{1}(T)$ and $g_{2}(T)$ with respect to **Y** and **Z** are:

(A.3) $$\partial g(Y,Z)=\nabla_{Y}g\partial Y+\nabla_{Z}g\partial Z=0$$

Define:

(A.4) $$\text{Jacobian matrix }J=\nabla_{Y}g=\left(\begin{array}{c} \nabla_{Y}g_{1}\\ \nabla_{Y}g_{2} \end{array}\right)$$

(A.5) $$\text{Control matrix }C=\nabla_{Z}g=\left(\begin{array}{c} \nabla_{Z}g_{1}\\ \nabla_{Z}g_{2} \end{array}\right)$$

Substituting (A.4) and (A.5) to (A.3), we have

(A.6) ∂g=J∂Y+C∂Z.

Given g(T)=0 and **J** is non-singular (the components of the vector **Y** must be selected such that **J** is non-singular), it follows that

(A.7) $$\partial Y=-J^{-1}C\partial Z$$

Substituting (A.7) to (A.2), the reduced gradient vector of *PF* with respect to the nonbasic variable **Z** is:

(A.8) $$\nabla_{r}\mathrm{PF}=\frac{\partial \mathrm{PF}}{\partial Z}=\nabla_{Z}\mathrm{PF}-\nabla_{Y}\mathrm{PF}J^{-1}C$$

Let $Y={\left(S_{1},S_{2}\right)}^{T}$, and $Z=T={\left(t_{1},t_{2},\ldots ,t_{M}\right)}^{T}$, we obtain the reduced gradient vector of *PF* with respect to **Z** (or **T**):

(A.9) $$\nabla_{Y}\mathrm{PF}=(\frac{\partial \mathrm{PF}}{\partial S_{1}},\frac{\partial \mathrm{PF}}{\partial S_{2}})=(0,0)$$

(A.10) $$\nabla_{z}\mathrm{PF}=(\frac{\partial \mathrm{PF}}{\partial t_{1}},\frac{\partial \mathrm{PF}}{\partial t_{2}},\ldots ,\frac{\partial \mathrm{PF}}{\partial t_{M}})=(p_{1}k_{1}e^{-k_{1}t_{1}},p_{2}k_{2}e^{-k_{2}t_{2}},\ldots ,p_{M}k_{M}e^{-k_{M}t_{M}})$$

(A.11) $$J=\left(\begin{array}{c} \begin{array}{cc} \frac{\partial g_{1}}{\partial S_{1}}\; & \frac{\partial g_{1}}{\partial S_{2}} \end{array}\\ \begin{array}{cc} \frac{\partial g_{2}}{\partial S_{1}}\; & \frac{\partial g_{2}}{\partial S_{2}} \end{array} \end{array}\right)=\left(\begin{array}{c} \begin{array}{cc} 1\; & 0 \end{array}\\ \begin{array}{cc} 0\; & 1 \end{array} \end{array}\right),\;\text{thus},\;J^{-1}=\left(\begin{array}{c} \begin{array}{cc} 1\; & 0 \end{array}\\ \begin{array}{cc} 0\; & 1 \end{array} \end{array}\right)$$

(A.12) $$C=\left(\begin{array}{ccc} \frac{\partial g_{1}}{\partial t_{1}}\; & \frac{\partial g_{1}}{\partial t_{2}},\ldots ,\; & \frac{\partial g_{1}}{\partial t_{M}}\\ \frac{\partial g_{2}}{\partial t_{1}}\; & \frac{\partial g_{2}}{\partial t_{2}},\ldots ,\; & \frac{\partial g_{2}}{\partial t_{M}} \end{array}\right)=\left(\begin{array}{ccc} 1\; & 1,\ldots ,\; & 1\\ d_{1}\; & d_{2},\ldots ,\; & d_{M} \end{array}\right)$$

Substituting (A.9)-(A.12) to (A.8), the reduced gradient vector of *PF* is:

(A.13) $$\nabla_{r}\mathrm{PF}=(p_{1}k_{1}e^{-k_{1}t_{1}},p_{2}k_{2}e^{-k_{2}t_{2}},\ldots ,p_{M}k_{M}e^{-k_{M}t_{M}})$$

Extreme point $T^{⁎}$ is obtained through a number of iterations where **T** is increased from **0** to $T^{⁎}$ in the search direction governed by $\nabla_{r}\mathrm{PF}$. Element $t_{i}$ of **T** is increased in the order of the value of $p_{i}k_{i}e^{-k_{i}t_{i}}$, subject to constraints $g_{1}$ and $g_{2}$ are satisfied. The higher the value of $p_{i}k_{i}e^{-k_{i}t_{i}}$, the higher the priority hence $t_{i}$ in increased.
